# Effects of cutaneous administration of an over-the-counter menthol cream during temperate-water immersion for exercise-induced hyperthermia in men

**DOI:** 10.3389/fphys.2023.1161240

**Published:** 2023-05-10

**Authors:** Gang Wang, Chansol Hurr

**Affiliations:** ^1^ Integrative Exercise Physiology Laboratory, Department of Physical Education, Jeonbuk National University, Jeonju, Republic of Korea; ^2^ Department of Physical Education, Xinyang Normal University, Xingang, China

**Keywords:** hyperthermia, skin cooling, topical analgesics, core temperature, cutaneous vasodilation, menthol

## Abstract

**Introduction:** Hyperthermia impairs various physiological functions and physical performance. We examined the effects of cutaneous administration with an over-the-counter (OTC) analgesic cream containing 20% methyl salicylate and 6% L-menthol during temperate-water immersion (TWI) for exercise-induced hyperthermia.

**Methods:** In a randomized crossover design, twelve healthy males participated in both of two experiments. Firstly, participants underwent a 15-min TWI at 20°C with (CREAM) or without (CON) cutaneous application of an analgesic cream. Cutaneous vascular conductance (CVC) was measured using laser doppler flowmetry during TWI. In a subsequent experiment, same participants performed a 30-min strenuous interval exercise in a heated (35°C) environment to induce hyperthermia (~39°C), which was followed by 15 min of TWI.

**Results:** Core body temperature, as measured by an ingestible telemetry sensor, and mean arterial pressure (MAP) were measured. CVC and %CVC (% baseline) were higher during TWI in CREAM than in CON (Condition effect: *p* = 0.0053 and *p* = 0.0010). An additional experiment revealed that core body heat loss during TWI was greater in CREAM than in CON (Cooling rate: CON 0.070 ± 0.020 vs. CREAM 0.084°C ± 0.026°C/min, *p* = 0.0039). A more attenuated MAP response was observed during TWI in CREAM than in CON (Condition effect: *p* = 0.0007).

**Conclusion:** An OTC analgesic cream containing L-menthol and MS augmented cooling effects when cutaneously applied during TWI in exercise-induced hyperthermia. This was, at least in part, due to the counteractive vasodilatory effect of the analgesic cream. The cutaneous application of OTC analgesic cream may therefore provide a safe, accessible, and affordable means of enhancing the cooling effects of TWI.

## 1 Introduction

Hyperthermia, a condition characterized by elevated body temperature, impairs various physiological functions and physical performance (Periard et al., 2021). Numerous studies have verified the effectiveness of a variety of cooling methods, including cold water immersion (CWI) (Proulx et al., 2003; Luhring et al., 2016), tarp-assisted cooling with oscillations (TACO) (Luhring et al., 2016; Hosokawa et al., 2017), application of a cooling garment (Abdallah et al., 2015; Xu et al., 2021), ice-sheet cooling (Butts et al., 2017), etc. Among these, CWI is currently regarded by numerous professional organizations as the gold standard treatment for hyperthermia (VanScoy et al., 2016; Belval et al., 2018).

External cold stimulation leads to vasoconstriction in the cutaneous microvasculature (Alba et al., 2019). This vasoconstricting response helps maintain a stable core temperature in the body as cutaneous blood flow is significantly dampened in response to external cold exposure, thereby decreasing the amount of heat transferred from core to skin (Charkoudian, 2010; Tansey and Johnson, 2015). In the case of hyperthermia, however, the cold-induced vasoconstriction in the skin could be a restriction factor, as the effect of external cooling can be partly limited to the cooled regions via conduction while heat exchanged between skin and core via the circulatory system is reduced (Taylor et al., 2008). Regarding this, the cooling effect of temperate-water immersion (TWI) at 26°C has been shown to be comparable to that of CWI at 14°C because of the maintenance of a higher peripheral blood flow during TWI (Taylor et al., 2008). Proulx et al. have also reported no differences in the cooling rate between the immersions at 8, 14, and 20°C for treating exercise-induced hyperthermia (Proulx et al., 2003). These studies suggest that TWI could be a safe, comfortable, and effective cooling modality for treating hyperthermic individuals. Also, there is potential that the cooling effects of TWI would be further augmented by overcoming the cold-induced cutaneous vasoconstriction associated with the immersion.

For decades, topical analgesic products have been utilized by sportspersons to relieve pain in local muscles and joints. The two main ingredients of over-the-counter (OTC) analgesics are L-menthol (1%–10%) and methyl salicylate (MS) (12%–30%) (Martin et al., 2004; Higashi et al., 2010). These two ingredients have a vasodilatory effect in the skin (Green and Flammer, 1989; Dolen et al., 2015; Craighead and Alexander, 2016; Craighead et al., 2017). Moreover, they have a synergistic effect on skin absorption, such that MS is more efficiently absorbed into the cutaneous vasculature in the presence of L-menthol (Yano et al., 1991). Our laboratory recently showed that the cutaneous application of analgesic cream can attenuate cold-induced vasoconstriction in the skin and potentially promote heat loss during hyperthermia (Wang et al., 2022). However, the effectiveness of OTC analgesic application administered with TWI as part of a hyperthermia treatment remains unknown.

In this paper, we determined whether an OTC analgesic cream containing L-menthol and MS augmented cooling effects when applied in TWI for treatment exercise-induced hyperthermia. We first tested whether the application of analgesic cream attenuated cold-induced vasoconstriction (*i.e.*, counteractive vasodilation) during TWI and compared this result to a control TWI. We then tested whether analgesic cream accelerated a reduction in core body temperature during TWI for treatment of exercise-induced hyperthermia.

## 2 Materials and methods

### 2.1 Ethical approval and participants

The study was conducted in accordance with the Declaration of Helsinki (2013) and all study procedures used in the current experiment were approved by the Institutional Review Board (IRB) at Jeonbuk National University (IRB #: JBNU 2022-01-004-002). All participants were verbally informed of the risks and discomforts associated with experimental trials, and written informed consent was obtained from all participants prior to their participation.

Twelve healthy males (age: 26.8 ± 2.6 years, height: 177.9 ± 5.4 cm, body weight: 75.7 ± 7.9 kg, BMI: 23.4 ± 1.6 kg ∙ m^-2^, body fat: 12.3% ± 3.9%) participated in this study. Participants with a history of genetic, cardiovascular, metabolic, or respiratory disease, allergic reactions to cold stimulation or topical analgesic cream, or a history of smoking, were excluded from participation. No participants reported taking medicines or supplements.

### 2.2 Overall experimental procedures

**FIGURE 1:**
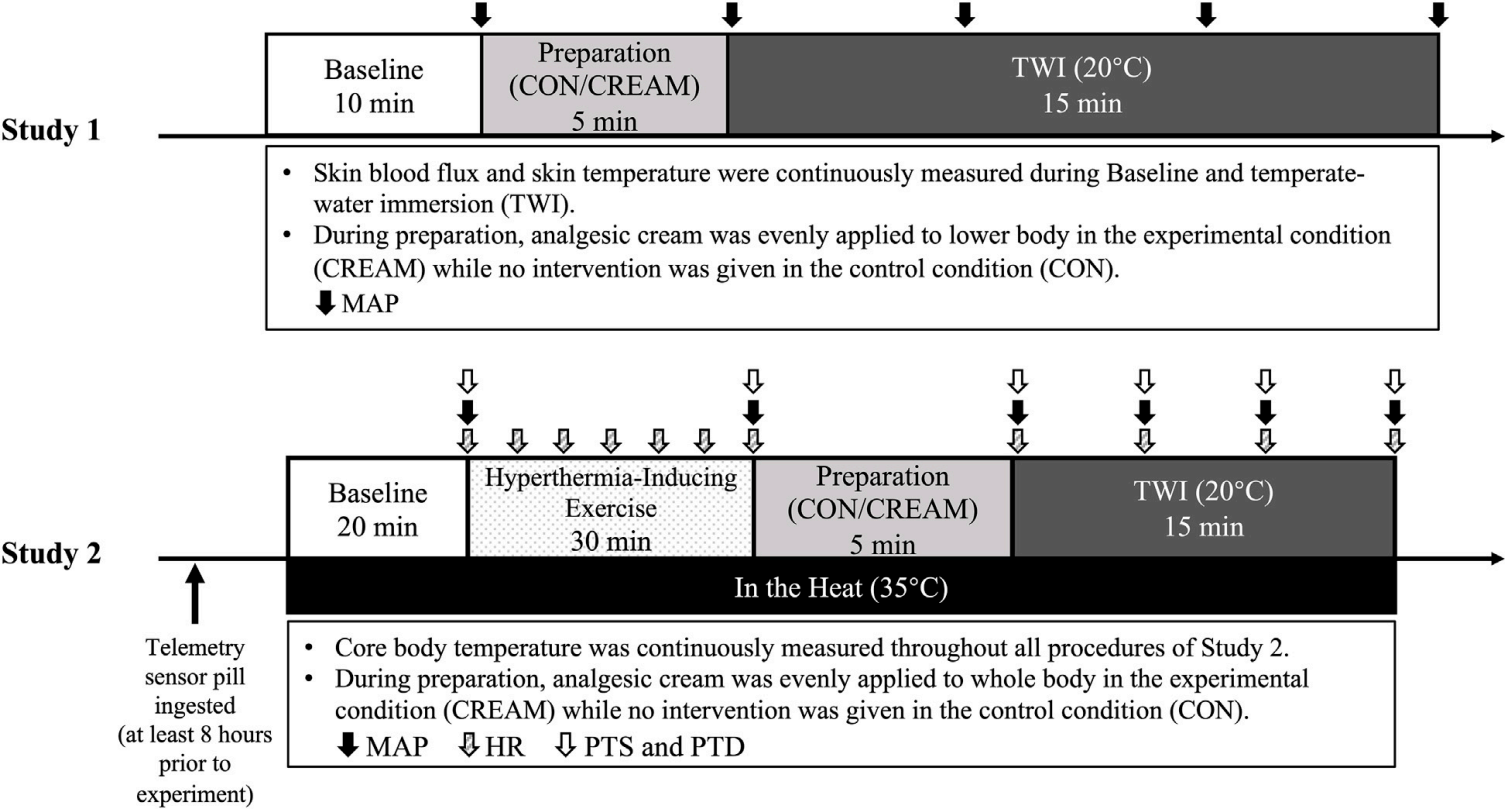
Experimental Protocols. Experimental procedures of two studies (Study 1 and 2). CON, a control condition; CREAM, an experimental condition that received cutaneous application of analgesic cream; TWI, temperate-water immersion at 20°C; MAP, mean arterial pressure; HR, heart rate; PTD, perceived thermal sensation; PTD, perceived thermal discomfort. Study 1 was performed in a thermoneutral environment (∼23°C, 50% humidity) while all procedures in Study 2 were conducted under hyperthermic conditions (35°C, 50% humidity).

A schematic experimental protocol is shown in Figure 1. During their first visit, participants were familiarized with the experimental protocol and instrumentation after providing written and verbal consent. Body mass (kg) and body fat (%) were measured (InBody 720, InBody Co., Seoul, South Korea) during the first visit. Each participant subsequently visited the laboratory a total of four times in the morning (7–10 a.m.) with at least 72-h separation between visits, two visits for Study 1 and two visits for Study 2. The order of each of the two visits within Study 1 and 2 was randomly assigned (*i.e.*, a randomized crossover design). Thus, all participants completed both Study 1 and 2. Participants were instructed to refrain from strenuous exercise, alcohol, and caffeine consumption during the 24 h prior to all visits and were asked to fast overnight for the ∼12 h preceding the visit. Participants were also asked to wear the same clothes (short-sleeved T-shirt and tight swim shorts) during all four visits.

#### 2.2.1 (Study 1) experiment procedures and measurements

All procedures in Study 1 were conducted in a temperature-controlled laboratory (∼23°C and 50% humidity). On the experimental day, participants were instructed to rest in a patient bed in a semi-supine position for 10 min (baseline). During the baseline period, two laser doppler probes (VP7 A/T with VMS-LDF2; Moor Instruments, Wilmington, DE, United States of America), wrapped with transparent plastic bags as waterproofing, were placed on the anterior thigh (15 cm apart to minimize inter-probe interaction). A line was drawn from the anterior superior iliac spine to the superior border of the patella. Two laser doppler flowmetry (LDF) probes were then placed in two spots from the center of the line of both thighs. Using LDF, skin blood flux and skin temperature (T_sk_) were recorded and averaged during the last minute of the baseline period. This was followed by three measurements of intermittent blood pressure via automated brachial auscultation (GE S/5 Light Patient Monitor; Datex-Ohmeda, Madison, WI, United States of America). Mean arterial pressure (MAP) was determined as one-third pulse pressure plus diastolic blood pressure. Cutaneous vascular conductance (CVC) was calculated as the ratio of skin blood flux to MAP.

Following the baseline measurement, the LDF probes were removed and analgesic cream was evenly applied to the entirety of the skin surface of both legs (CREAM), which was followed by re-attachment of the probes. For the control condition (CON), probes were removed and re-attached without administration of analgesic cream. In our pilot study, SkBF readings were neither affected by the re-attachment of the laser doppler probes nor the transparent plastic bag for waterproofing (data not shown). Participants were then immersed in a bathtub (498L) in which the water temperature was maintained at 20°C (temperate-water immersion, TWI) to the iliac crest for 15 min (Mawhinney et al., 2017). During the 15-min TWI period, skin blood flux and T_sk_ were continuously measured, and intermittent blood pressure was measured three times every 5 min. It should be noted that, rather than whole body immersion, TWI was performed only for the lower body in Study 1 as a result of technical difficulties associated with laser doppler measurement in the water (*i.e.*, waterproofing issue).

##### 2.2.1.1 Cutaneous application of topical analgesic cream

The OTC analgesic cream that was used in our study consisted of 6% L-menthol, 20% methyl salicylate (MS), and other ingredients, including lanolin and mineral oil, sorbitanmonostearate (surfactant), polysorbate 60 (surfactant), trolamine, and purified water (Antiphlamine-S, Yuhan, South Korea). In a previous study, we confirmed that dose of 0.64 μ L/cm^2^ placed on the skin surface exhibited a potent vasodilatory effect (increasing blood flux by 80%–115% relative to baseline) over the course of 1 h (Wang et al., 2022).

#### 2.2.2 (Study 2) experimental procedures and measurements

Based on findings in Study 1, we performed Study 2 to assess whole body heat loss. Along with overnight fasting, participants were asked to ingest a telemetry sensor pill (CorTemp Ingestible Core Body Temperature Sensor, HQ, Palmetto, FL, United States) to measure core body temperature (T_CORE_) at least 8 h before the trial to avoid temperature deviation due to stomach contents (Domitrovich et al., 2010). Upon arrival, a core temperature recorder (CorTemp Data Recorder, HQ, Palmetto, FL, United States) was wirelessly connected with the sensor pill and placed on the back of participants to allow for continuous recording of their core temperature. Participants drank 200 mL of thermoneutral water (37°C) to minimize interference during the core temperature reading and no additional water was allowed afterwards. Subjects entered the temperature-controlled room which was maintained at 35°C (∼50% humidity). All procedures conducted in Study 2 were completed in a hot environment (Figure 1).

Following a 20-min baseline period in the sitting position (baseline), participants performed a 30-min regimen of strenuous interval exercise that consisted of 30 s each of jumping jacks, high knees, squat, lunges, and cycling with 5 s breaks given between each activity. During this exercise protocol, T_CORE_ increased by 2.02°C ± 0.18°C from the Baseline (Baseline 37.02 ± 0.14 vs. Post Exercise 39.04°C ± 0.15°C for the two conditions combined). Based on the age-predicted exercise intensity (heart rate reserve or HRR) (Lui et al., 2011), the exercise intensity in the current study was between 75% and 85%.

Once hyperthermia was induced, participants removed all clothes except for the tight swim shorts and toweled off their sweat. They then were immersed in a recumbent position in a 20°C water bathtub (498L) without (CON) or with (CREAM) the analgesic cream administered over the entire surface of the legs and arms as well as torso (0.64 μ L/cm^2^ of skin surface), which took approximately less than 1 min. During the 15 min of TWI, participants completely submerged their bodies up to the sternum. The water temperature was measured every 3 min and was maintained by adding an appropriate amount of ice-water and then stirring slightly.

Perceived thermal sensation (Brazaitis et al., 2014), perceived thermal discomfort (Mekjavic et al., 2021), and blood pressure (BP) were measured at the end of the baseline and exercise, at the onset and throughout TWI (every 5 min), and post TWI. The thermal sensation scale ranged from 1 (very cold) to 9 (very hot), with 5 being neutral. The scale of perceived thermal discomfort ranged from 1 (neutral) to 5 (extreme discomfort).

### 2.3 Statistical analysis

Data are expressed as mean ± SD. Statistical significance was set as *p* < 0.05. The assumption of normality was verified using the Shapiro-Wilks W-Test. Variables were analyzed using two-way repeated-measures ANOVA, which was followed by Šidák’s multiple comparisons; the factors were intervention conditions (CON/CREAM) and time. A two-tailed paired *t*-test was used to determine a statistical difference in cooling rates between the conditions. Cohen’s d effect size (ES) was reported (ES > 0.2, small; >0.5, moderate; >0.8, large) when statistical differences were found between conditions. Statistical analyses were performed using GraphPad Prism 9.2.0 software (GraphPad, La Jolla, CA, United States).

## 3 Results

### 3.1 Study 1

#### 3.1.1 Cutaneous vascular conductance (CVC), % CVC, and skin temperature (T_sk_)

**FIGURE 2:**
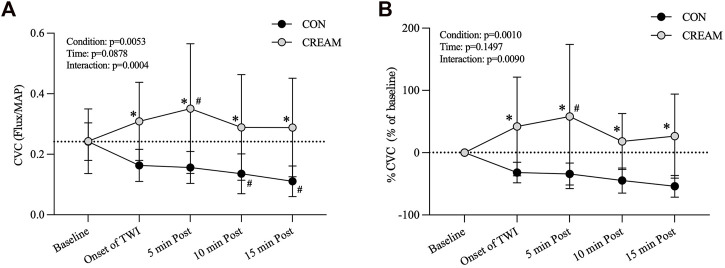
Cutaneous Vascular Conductance (CVC) and % CVC. Changes in CVC **(A)** and % CVC **(B)** during Baseline and 15-min TWI. CON, a control condition; CREAM, an experimental condition that received cutaneous application of analgesic cream. Two-way repeated measures ANOVA with a Šidák’s *post hoc* test for all. Data are presented as the mean ± SD (*n* = 12). *p < 0.05 between the conditions. #*p* < 0.05 vs. baseline of each condition.

During the 15-min TWI, CVC in CREAM remained elevated at levels higher than those seen in CON (Condition effect: *p* = 0.0053) (Figure 2A). Also, a significant interaction (condition × time) was seen (*p* = 0.0004). From the *post hoc* analyses, CVC was elevated from the onset of TWI to the end of TWI (Onset of TWI: *p* = 0.0001, ES = 1.67; 5 min Post: *p* < 0.0001, ES = 1.52; 10 min Post: *p* < 0.0001, ES = 1.33; 15 min Post: *p* < 0.0001, ES = 1.74) (Figure 2A). CVC was lower in CON relative to the baseline during the TWI (10 min Post: *p* = 0.0112, ES = 1.73; 15 min Post: *p* = 0.0009, ES = 2.43) while an increased CVC was observed in CREAM (5 min Post: *p* = 0.0095, ES = 0.70).

Similarly, % CVC was higher in CREAM relative to CON (Condition effect: *p* = 0.0010) (Figure 2B). A significant interaction was found (*p* = 0.0090). % CVC was greater in CREAM from the onset of TWI to the end of TWI (Onset of TWI: *p* = 0.0012, ES = 1.61; 5 min Post: *p* < 0.0001, ES = 1.45; 10 min Post: *p* = 0.0071, ES = 2.02; 15 min Post: *p* = 0.0004, ES = 1.98) (Figure 2B). % CVC was increased in CREAM relative to the baseline during the TWI (5 min Post: *p* = 0.0287, ES = 1.05) but no significant differences were observed in CON (*p* > 0.05 for all).

T_sk_ decreased similarly in both conditions during TWI (Time and Condition effect: *p* < 0.0001 and 0.6455, respectively). No differences in T_sk_ were found between conditions during TWI (Onset of TWI: *p* > 0.9999; 5 min Post: *p* = 0.7019; 10 min Post: *p* = 0.7129; 15 min Post: *p* = 0.8322) (Table 1).

**TABLE 1:** Changes in Skin Temperature. Skin temperature (°C) during baseline and 15-min TWI. CON, a control condition; CREAM, an experimental condition that received cutaneous application of analgesic cream. Two-way repeated measures ANOVA with a Šidák’s *post hoc* test for all. Data are presented as the mean ± SD (*n* = 12). No significant differences were found between the conditions.

	Baseline	TWI (min)			
		Onset	5	10	15
CON	30.75 ± 0.92	23.14 ± 0.99	21.59 ± 0.52	21.51 ± 0.69	21.67 ± 0.77
CREAM	30.44 ± 1.06	23.17 ± 1.12	21.92 ± 0.94	21.83 ± 0.88	21.94 ± 0.82

### 3.2 Study 2

#### 3.2.1 Core temperature (T_CORE_) and cooling rate

**FIGURE 3:**
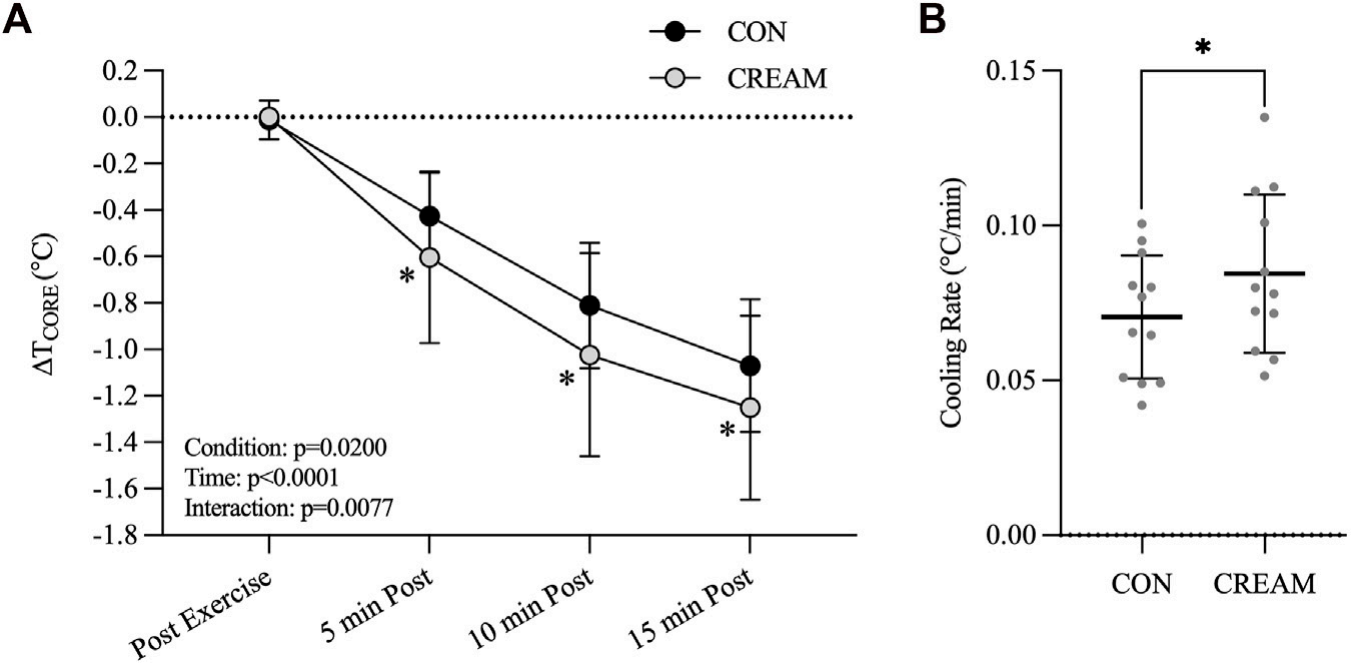
Core Temperature and Cooling Rate. Changes in T_CORE_ ( ∆ T_CORE_) **(A)** and cooling rate **(B)** during 15-min TWI. CON, a control condition; CREAM, an experimental condition that received cutaneous application of analgesic cream. Two-way repeated measures ANOVA with a Šidák’s *post hoc* test and a paired *t*-test was used for **(A and B)**, respectively. Data are presented as the mean ± SD (*n* = 12). *p < 0.05 between the conditions.

The baseline T_CORE_ was similar between conditions (CON: 37.02 ± 0.15 vs. CREAM: 36.95°C ± 0.14°C, *p* = 0.8763) (Figure 3A). During the 30-min exercise, T_CORE_ increased similarly in both conditions (Baseline: 37.02 ± 0.15 vs. Post Exercise: 39.04°C ± 0.15°C, Time effect: *p* < 0.0001, Condition effect: *p* = 0.5066) with no differences observed between conditions by the end of exercise (Post Exercise) (CON: 39.03 ± 0.16 vs. CREAM: 39.04°C ± 0.15°C, *p* = 0.598).

During 15-min TWI, a decline in T_CORE_ ( ∆ T_CORE_) was observed in both conditions (Time effect: *p* < 0.0001), though an even a greater reduction in T_CORE_ was detected in CREAM (Condition effect: *p* = 0.0200; 5 min Post: CON − 0.425 ± 0.186 vs. CREAM − 0.604°C ± 0.369°C, *p* = 0.0027, ES = 0.67; 10 min Post: CON − 0.811 ± 0.270 vs. CREAM − 1.024°C ± 0.438°C, *p* = 0.0003, ES = 0.63; 15 min Post: CON − 1.070 ± 0.286 vs. CREAM − 1.252°C ± 0.396°C, *p* = 0.0022, ES = 0.56) (Figure 3A). A significant interaction was found (Condition × Time) (*p* = 0.0077).We also analyzed the cooling rate during the 15-min TWI; this was higher in CREAM relative to CON (CON 0.070 ± 0.020 vs. CREAM 0.084°C ± 0.026°C/min, *p* = 0.0039, ES = 0.65) (Figure 3B).

#### 3.2.2 Blood pressure and heart rate (HR)

**FIGURE 4:**
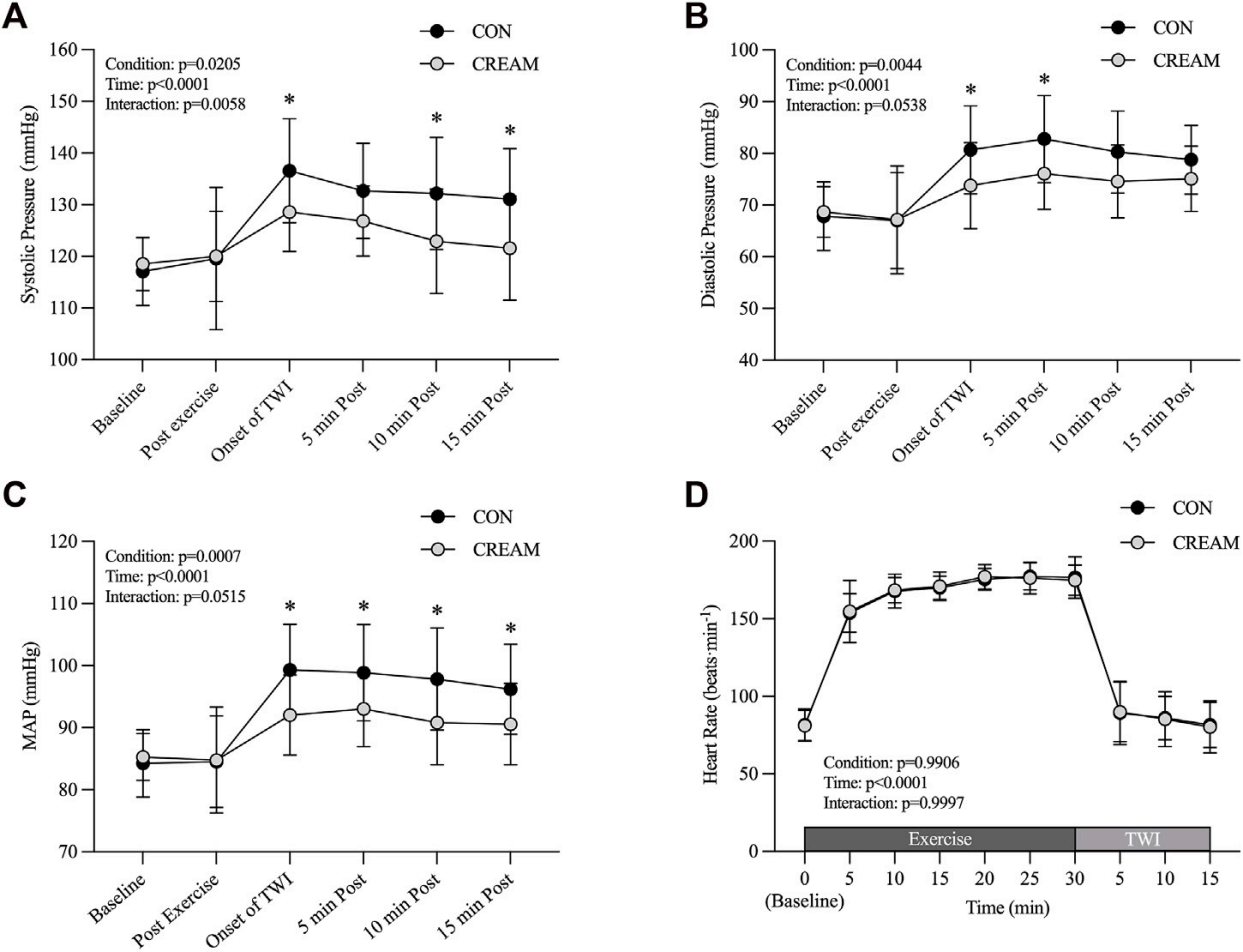
Blood Pressure. Changes in systolic blood pressure (SBP) **(A)**, diastolic blood pressure (DBP) **(B)**, mean arterial pressure (MAP) **(C)**, and heart rate (HR) **(D)** during baseline, post-exercise, and 15-min TWI. CON, a control condition; CREAM, an experimental condition that received cutaneous application of analgesic cream. Two-way repeated measures ANOVA with a Šidák’s *post hoc* test for all. Data are presented as the mean ± SD (*n* = 12). *p < 0.05 between the conditions.

During the TWI, a reduced systolic blood pressure (SBP) was observed in CREAM relative to CON (Condition effect: *p* = 0.0205, Onset of TWI: CON 136.58 ± 10.05 vs. CREAM 128.58 ± 7.65 mmHg, *p* = 0.015, ES = 0.94; 5 min Post: CON 132.67 ± 9.19 vs. CREAM 126.83 ± 6.78 mmHg, *p* = 0.1397; 10 min Post: CON 132.17 ± 10.85 vs. CREAM 122.92 ± 10.09 mmHg, *p* = 0.0034, ES = 0.92; 15 min Post: CON 131.08 ± 9.77 vs. CREAM 121.58 ± 10.07 mmHg, *p* = 0.0025, ES = 1.00) (Figure 4A). Also, significant time effect and interaction (Condition × Time) were found (Time effect: *p* < 0.0001; Interaction: *p* = 0.0058).

We also noted a trend towards lowered diastolic blood pressure (DBP) during TWI (Condition effect: *p* = 0.0044, Onset of TWI: CON 80.67 ± 8.51 vs. CREAM 73.75 ± 8.30 mmHg, *p* = 0.0186, ES = 0.86; 5 min Post: CON 82.75 ± 8.44 vs. CREAM 76.08 ± 6.91 mmHg, *p* = 0.0254, ES = 0.91; 10 min Post: CON 80.25 ± 7.93 vs. CREAM 74.58 ± 7.06 mmHg, *p* = 0.0822; 15 min Post: CON 78.75 ± 6.62 vs. CREAM 75.08 ± 6.29 mmHg, *p* = 0.4925) (Figure 4B). A significant time effect (Time effect: *p* < 0.0001) was detected while no significant interaction (Condition × Time) was found (*p* = 0.0538).

In both conditions, MAP significantly increased during TWI (Time effect: *p* < 0.0001). Interestingly, MAP was lower in CREAM than in CON during TWI (Condition effect: *p* = 0.0007) (Onset of TWI: CON 99.31 ± 7.36 vs. CREAM 92.03 ± 6.44 mmHg, *p* < 0.0001, ES = 1.10; 5 min Post: 98.86 ± 7.76 vs. CREAM 93.00 ± 6.05 mmHg, *p* = 0.0028, ES = 0.89; 10 min Post: CON 97.83 ± 8.24 vs. CREAM 90.81 ± 6.80 mmHg, *p* = 0.0051, ES = 0.98; 15 min Post: CWI 96.19 ± 7.25 vs. 90.58 ± 6.58 mmHg, *p* = 0.0495, ES = 0.85) (Figure 4C). No significant interaction (Condition × Time) was found (*p* = 0.0515).

No differences in HR were seen during baseline between conditions (Condition effect: *p* = 0.9906). Both conditions showed similar increases in HR during the 30-min exercise, and ultimately no discrepancy was seen by the end (*p* > 0.9999 for all time points during exercise). HR also declined significantly in both conditions with no noticeable differences between them (*p* > 0.9999 for all time points during TWI (Figure 4D).

#### 3.2.3 Perceived thermal sensation (PTS) and perceived thermal discomfort (PTD)

PTS was similar between conditions during baseline (*p* = 0.9872), post exercise (*p* = 0.9872), and TWI (Onset of TWI: *p* = 0.9106; 5 min Post: *p* = 0.4964; 10 min Post: *p* = 0.9997; 15 min Post: *p* = 0.9872) (Table 2). Similarly, no differences in PTD were observed between conditions during baseline (*p* = 0.6234), post exercise (*p* > 0.9999), and TWI (Onset of TWI: *p* = 0.9974; 5 min Post: *p* > 0.9999; 10 min Post: *p* = 0.9974; 15 min Post: *p* = 0.9135) (Table 2).

**TABLE 2:** Perceived Thermal Sensation (PTS) and Perceived Thermal Discomfort (PTD). PTS (score range: 1–9) and PTD (score range: 1–5) during baseline, post-exercise, and 15-min TWI is shown. CON, a control condition; CREAM, an experimental condition with cutaneous application of analgesic cream. Two-way repeated measures ANOVA with a Šidák’s *post hoc* test for all. Data are presented as the mean ± SD (*n* = 12). No significant differences were found between the conditions.

		Baseline	Post exercise	TWI (min)			
				Onset	5	10	15
PTS	CON	6.17 ± 0.39	8.83 ± 0.39	1.50 ± 0.79	1.75 ± 0.87	1.75 ± 1.06	1.83 ± 1.03
	CREAM	6.33 ± 0.49	8.67 ± 0.49	1.25 ± 0.62	1.33 ± 0.49	1.67 ± 0.65	1.67 ± 0.65
PTD	CON	1.50 ± 0.52	3.75 ± 0.45	3.75 ± 0.45	3.42 ± 0.67	3.42 ± 0.67	3.33 ± 0.49
	CREAM	1.75 ± 0.45	3.75 ± 0.45	3.83 ± 0.39	3.42 ± 0.52	3.50 ± 0.52	3.17 ± 0.39

## 4 Discussion

We investigated the effects of an OTC analgesic cream containing 20% methyl salicylate (MS) and 6% L-menthol administered cutaneously during TWI at 20°C as a treatment for exercise-induced hyperthermia. In Study 1, a significant vasoconstriction in the skin occurred during a control TWI (a decrease by 32%–59% from baseline); and a counteractive vasodilatory effect was observed during the TWI after the OTC analgesic cream was applied to the skin, such that cutaneous vascular conductance (CVC) was higher than the baseline counterpart (by 19%–44%) (Figure 2A). Skin temperature (T_sk_) dropped in both conditions during the TWI with no difference seen between conditions (Table 1).

Relying on our findings in Study 1, we performed a subsequent experiment (Study 2) and found that cutaneous administration of analgesic cream promoted core body heat loss during TWI administered in response to exercise-induced hyperthermia (Figures 3A, B). Together, the analgesic cream improved the cooling effect of TWI potentially via vasodilation that counteracted the cold-induced vasoconstriction. Interestingly, we also observed that the analgesic cream lowered the blood pressure during the TWI (Figure 4).

Acceptable cooling rates for treating hyperthermic condition are >0.08°C/min with an ideal rate of cooling being >0.16°C/min (McDermott et al., 2009). Thus, the proposed cooling method in our study (CON: 0.070°C ± 0.020°C/min and CREAM: 0.084°C ± 0.026°C/min) would not be the best options for individuals with heat-related illnesses such as exertional heat stroke (EHS) although the proposed TWI (*i.e.*, CREAM) did meet ‘minimal’ guideline for EHS treatment (>0.08°C/min). In athletic competition, however, athletes may not have adequate time to cool between breaks in competition, which would be ∼10 min at most between halves or quarters. In our study, cooling rates during the first 5-min of TWI were 0.121°C ± 0.061°C/min in CREAM while a control TWI showed 0.085°C ± 0.036°C/min. Therefore, an OTC analgesic cream administered during TWI would be applicable for those who have limited time for cooling.

Cold-induced vasoconstriction is mediated by a variety of intrinsic mechanisms including norepinephrine (NE) synthesis and release, adrenergic receptors, nitric oxide (NO), as well as activation of Rho-associated kinase (ROCK) signaling mechanisms (Lang et al., 2009; Alba et al., 2019). Our data revealed that cutaneous vasoconstriction during TWI was clearly lower in CREAM from Study 1, such that skin blood flux or CVC were 2-3 times higher in CREAM than in CON during the TWI. Considering the main ingredients in our analgesic cream (and in the majority of OTC analgesic products), we speculate that the pharmacological actions of MS and L-menthol may play an important role in decreasing cold-induced vasoconstriction during TWI.

MS, a common ingredient of topical analgesics, is a non-steroidal anti-inflammatory drug (NSAID) made from natural plant extracts. As an agonist for transient receptor potential vanilloid subtype 1 (TRPV1), MS is involved in the nociceptive signaling of sensory nerves (Ohta et al., 2009). A recent study showed that TRPV1 mediates thermoregulation, such that inhibition of TRPV1 activates sympathetic nervous activity (SNA) and associated heat gaining responses such as cutaneous vasoconstriction (Alawi et al., 2015). Cold stimulation is known to suppress TRPV1 activation (Chung and Wang, 2011), which may partly explain the cold-induced thermogenic responses observed in the human body. Thus, MS that binds to TRPV1 may blunt sympathetic-induced vasoconstriction despite the fact that cold stimulation concomitantly suppresses TRPV1. The opposite actions for TRPV1 might explain the blunted cutaneous vasoconstriction we observed in the CREAM during TWI. In addition, the TRPV1 expressed in endothelial cells lead to increased endothelial NO synthase (eNOS) phosphorylation and NO production (Wang et al., 2017), which may also explain the counteractive vasodilation in the skin of the CREAM as we observed during the TWI in our study. However, at this point these proposed mechanisms, as an explanation for this phenomenon, are merely speculative and future studies are needed to verify the mechanisms.

L-menthol is a known vasodilator that operates through eNOS, an L-type voltage-gated calcium blockade, and endothelium-derived hyperpolarizing factor (EDHF) (see review paper by Silva (Silva, 2020)). L-menthol also suppresses the reactive oxygen species (ROS)-induced RhoA/ROCK pathway via transient receptor potential melastatin 8 (TRPM8) channels (Fernandez-Tenorio et al., 2011; Xiong et al., 2017), a signaling pathway associated with cold-induced vasoconstriction. Along with its vasodilatory effects, L-menthol may blunt the increased activation of the RhoA/ROCK signaling induced by cold stimulation, and prompt the translocation of the α_2C_-adrenergic receptor (Chotani et al., 2000; Bailey et al., 2004). Two recent studies showed that L-menthol application triggers a sympathetic-mediated vasoconstriction that delays the cooling effects associated with CWI (*i.e.*, heat-conservation responses) (Kounalakis et al., 2010; Botonis et al., 2018). However, the analgesic cream used in our study consisted of a large amount of MS (20%), which complicates the comparison between their findings and ours. MS-induced TVPV1 activation may blunt sympathetic-mediated responses (Alawi et al., 2015) and inhibit sensory nerves and perception (Ohta et al., 2009). Although previous studies have reported a decrease in muscle and skin temperature in response to menthol application, possibly indicating menthol-induced vasoconstriction (Lasanen et al., 2016; Hunter et al., 2018; Gillis et al., 2021), the idea that the ethanol contained in a menthol product induces evaporative heat loss cannot be ruled out (Hunter et al., 2018). Nevertheless, it is obvious that the OTC analgesic product used in this current study resulted in an increase in cutaneous blood perfusion by 80%–115% from baseline, a potent vasodilating effect (Wang et al., 2022). Further data is needed to explain this discrepancy in menthol’s effects on vascular function during cooling.

An elevation in blood pressure is normally seen during skin cooling due to SNA-mediated peripheral vasoconstriction (Koehn et al., 2020), which indicates a potential risk to the cardiovascular system (Koehn et al., 2012; Tipton et al., 2017). It was also reported that medical professionals are sometimes hesitant to utilize whole-body CWI due to the fear of inducing shock (Hosokawa et al., 2019). Nevertheless, the majority of cooling studies have not reported such changes in blood pressure during cooling. In the current study, MAP increased in response to the 15-min TWI by 12–15 mmHg with systolic and diastolic pressure elevated by 12–17 mmHg and 12–14 mmHg, respectively. Intriguingly, participants cooled with analgesic cream experienced a lower blood pressure (Figures 4A–C). The effect size ranged from 0.85 to 1.10, so the blood pressure lowering effect of the analgesic cream seems clinically significant. It is well known that MS exerts an analgetic effect by desensitizing TRPV1 on sensory nerves (Ohta et al., 2009). Recent data has also revealed that TRPV1 plays a key role in the regulation of blood pressure via SNA modulation (Zhong et al., 2019). We accordingly speculate that cold-induced activation of sensory afferent nerves might have been blunted by MS-induced desensitization of TRPV1, which consequently attenuated reflex vasoconstriction and blood pressure responses during TWI. In addition to MS, L-menthol may have entered the systemic circulation and suppressed the RhoA/ROCK pathway, which has previously been observed in humans (Martin et al., 2004). Other studies have also shown that menthol supplementation can decrease blood pressure in hypertensive animals (Sun et al., 2014; Xiong et al., 2017).

With reference to the perceived thermal discomfort (PTD) data in our study, perceived discomfort during TWI was comparable to that measured post exercise (Table 2); TWI at 20°C was quite unpleasant. The water temperature of TWI was set between 20°C–26°C in previous investigations (Proulx et al., 2003; Taylor et al., 2008). It may be worth investigating the effects of TWI when a higher water temperature is used in combination with additional interventions that can improve the cooling effects of TWI. We speculate that this may result in a greater counteractive vasodilatory effect triggered by the analgesic cream, as warmer water would lead to a decrease in cold-induced SNA response and consequent peripheral vasoconstriction.

### 4.1 Limitations

There are some limitations in this study. Firstly, we were not able to measure CVC or %CVC during TWI in Study 2 because of technical difficulties associated with waterproofing LDF probes. To measure CVC during the whole-body water immersion, LDF probes should have been waterproofed for longer length (>50 cm). Thus, we chose to perform a separate experiment (lower body TWI of Study 1) that could only provide an indirect explanation of the greater cooling effect of TWI observed with the application of the analgesic cream in Study 2.

Although we observed a potent counteractive vasodilatory effect from the analgesic cream during TWI in Study 1, its cooling effect on a hyperthermic body did not seem to be clinically meaningful (Figure 3). This may be because Study 2 involved whole-body TWI, and therefore elicited greater SNA activation and vasoconstriction (*i.e.*, greater reflex vasoconstriction), while Study 1, which involved only the lower body, led to less cold-induced reflex responses. Supporting this theory, we observed no increases in blood pressure during the lower body TWI in Study 1 relative to blood pressure in the baseline period (data not shown).

Sweat loss is closely associated with thermoregulatory function (Periard et al., 2021). There is a possibility that sudomotor function might have been affected by the analgesic. However, in our recent study, there was no discrepancy in sweat loss between conditions (CON vs. CREAM) (Wang et al., 2022) and we did not measure sweat loss in the current study. Also, female participants were not included in the current study as a subcutaneous fat layer largely affects cooling efficiency (Boehm and Miller, 2019). Data interpretation of our study, therefore, should be limited to males and future studies with both sexes included are warranted.

We speculate that the TRPV1 and RhoA/ROCK signaling pathways are involved in mediating the beneficial effects in application of analgesic cream containing 20% MS and 6% L-menthol. However, an analgesic product that does not contain active ingredients (*i.e.*, a product without MS and L-menthol) may better serve as a control intervention. Future studies investigating the molecular mechanisms that underlay the counteractive vasodilatory effect of analgesic cream applied in response to external cold stimulation are also needed.

## 5 Perspectives

We determined that an OTC analgesic cream containing L-menthol and MS augments TWI’s cooling effects when applied in response to exercise-induced hyperthermia. The improved heat loss may be, at least in part, due to the counteractive vasodilatory effect of the analgesic cream. This tested cooling method also blunted the increased blood pressure normally observed during external cooling. A cutaneous application of OTC analgesic cream therefore suggests itself as a safe, accessible, and affordable means of improving the cooling effects of TWI.

## Data Availability

The original contributions presented in the study are included in the article/supplementary material, further inquiries can be directed to the corresponding author.
